# Screening and monitoring microbial xenobiotics’ biodegradation by rapid, inexpensive and easy to perform microplate UV-absorbance measurements

**DOI:** 10.1186/1756-0500-7-101

**Published:** 2014-02-22

**Authors:** Bastian Herzog, Hilde Lemmer, Harald Horn, Elisabeth Müller

**Affiliations:** 1 Chair of Urban Water Systems Engineering, Technische Universität München, Am Coulombwall, Garching D-85748, Germany; 2 Bavarian Environment Agency, Bürgermeister-Ulrich-Str. 160, Augsburg D-86179, Germany; 3 Karlsruhe Institute of Technology, Engler-Bunte-Institut, Bereich Wasserchemie und Wassertechnologie, Karlsruhe D-76131, Germany

**Keywords:** Xenobiotics, Biodegradation, Microplate measurement, UV-absorbance, Benzotriazoles, Sulfamethoxazole

## Abstract

**Background:**

Evaluation of xenobiotics biodegradation potential, shown here for benzotriazoles (corrosion inhibitors) and sulfamethoxazole (sulfonamide antibiotic) by microbial communities and/or pure cultures normally requires time intensive and money consuming LC/GC methods that are, in case of laboratory setups, not always needed.

**Results:**

The usage of high concentrations to apply a high selective pressure on the microbial communities/pure cultures in laboratory setups, a simple UV-absorbance measurement (UV-AM) was developed and validated for screening a large number of setups, requiring almost no preparation and significantly less time and money compared to LC/GC methods. This rapid and easy to use method was evaluated by comparing its measured values to LC-UV and GC-MS/MS results. Furthermore, its application for monitoring and screening unknown activated sludge communities (ASC) and mixed pure cultures has been tested and approved to detect biodegradation of benzotriazole (BTri), 4- and 5-tolyltriazole (4-TTri, 5-TTri) as well as SMX.

**Conclusions:**

In laboratory setups, xenobiotics concentrations above 1.0 mg L^-1^ without any enrichment or preparation could be detected after optimization of the method. As UV-AM does not require much preparatory work and can be conducted in 96 or even 384 well plate formats, the number of possible parallel setups and screening efficiency was significantly increased while analytic and laboratory costs were reduced to a minimum.

## Background

Evaluation and monitoring of the biodegradation potential of different activated sludge (AS) communities as well as other microbial systems is often time consuming and money intensive as mostly techniques like LC-UV, LC-MS/MS or GC-MS/MS for determination of concentrations of various compounds are used. When very low concentrations (ng L^-1^ or μg L^-1^) need to be measured these techniques are the only option, but in many laboratory biodegradation setups under standardized conditions much higher concentrations in the mg L^-1^ range are used. Furthermore, for pre-testing biodegradation potentials in a large number of setups, LC/GC methods require too much time and are often not necessary. In many screening experiments, knowing exact concentrations is not necessary as it is sufficient to know whether biodegradation occurs or not. Therefore a rapid, easy to use and inexpensive technique is required to screen a large number of different setups for their biodegradation potential towards different xenobiotics. In a research project benzotriazoles and the antibiotic sulfamethoxazole were used as xenobiotics to evaluate their biodegradation pattern in laboratory setups.

These xenobiotic compounds are polar micropollutants with a wide spectrum of use. Benzotriazoles are extensively used as corrosion inhibitors [1] while SMX is one of the most commonly used antibiotics to treat human infections [2,3]. Both compounds show high water solubility, an ubiquitary occurrence in almost all water bodies and an incomplete biological removal [4-11]. Former studies already proved that wastewater treatment plants (WWTP), which receive domestic and industrial wastewater, constitute one major point source for these compounds to be released into the aquatic environment [6,12-15]. Therefore biodegradation studies, performed under specific laboratory conditions to exclude abiotic processes, are implicitly required to gain information about the biological removal potential of activated sludge communities as they are one way to reduce the input of these compounds into aquatic environmental systems [16-20]. Laboratory experiments already proved a completely different removal behavior of benzotriazole, 4- and 5-tolyltriazole [11,21] but biodegradation conditions remain rather unclear. SMX, in contrast, showed sometimes an almost complete removal in lab-scale setups inoculated with AS communities and/or mixed cultures under different conditions tested [22,23]. Furthermore, only little information is available on individual organisms being capable of SMX biodegradation as well as biodegradation potential under different redox and nutrient conditions [24-27].

To address the need for a rapid screening, this study provides a simple and inexpensive method to evaluate the potential of AS communities, mixed bacterial pure culture communities as well as single pure culture bacteria to biodegrade benzotriazoles and SMX. A test system for biodegradation detection that requires almost no preparation, uses simple UV absorbance measurements (UV-AM) and can be done in microplate setups, was developed and evaluated by comparing its results with LC-UV and GC-MS/MS. That system allows screening a large number of setups, minimizing laboratory costs and experimental time.

## Results and discussion

### Evaluation of UV-AM

Evaluation of the UV-AM method was performed regarding the following aspects: a) fate of the parent substances by monitoring the change in absorbance due to removal, b) screening for potential transformation products with spectral scans and c) optimization of cultivation media to meet the requirements for application in UV-AM.

### Parent substances

**Figure 1:**
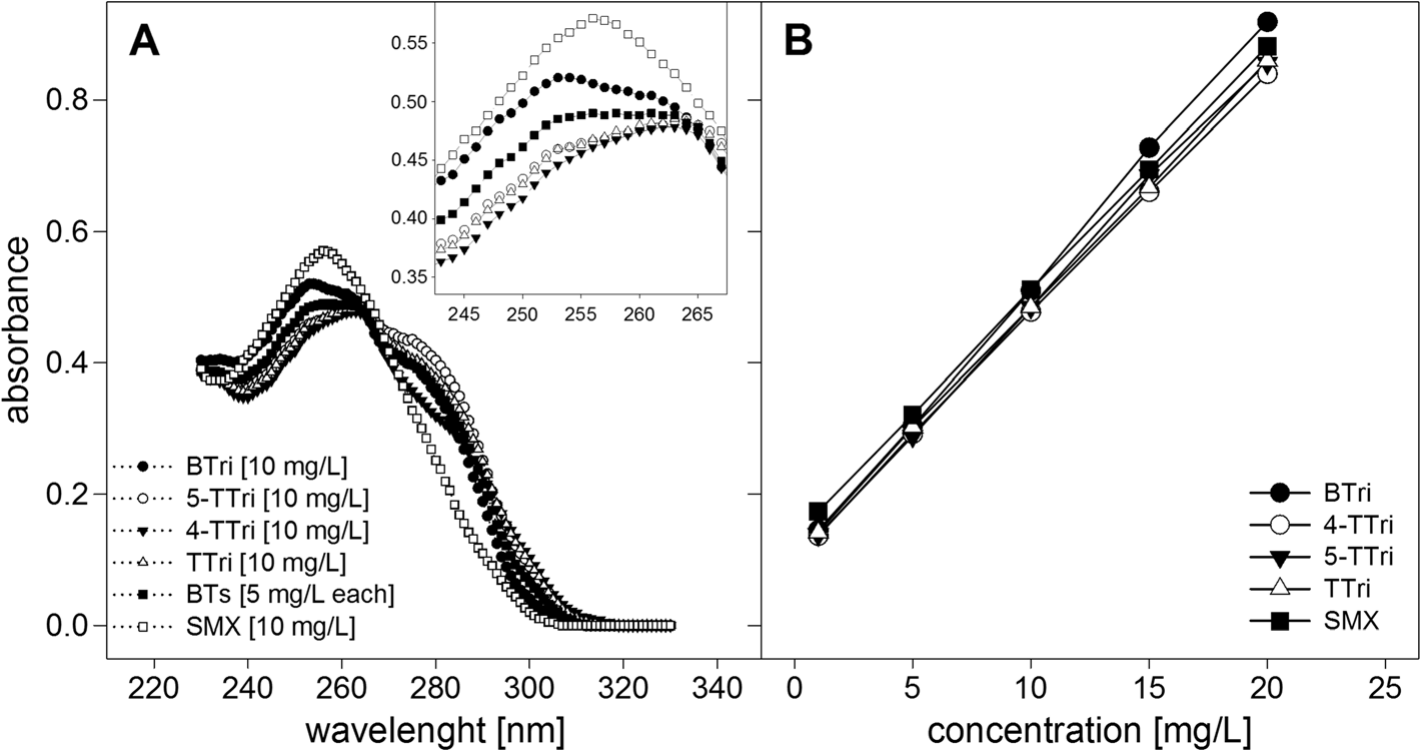
**A and B UV-absorbance spectra of tested xenobiotic compounds. A)** in pure water at 10 mg L^-1^. Absorbance maxima are given in Table 1. **B)** Calibration curves of BTri, 4- and 5-TTri, TTri and SMX in pure water. Concentrations were 1.0, 5.0, 10.0, 15.0 and 20.0 mg L^-1^. Absorbance was measured at 257 nm (SMX) and 262 nm (benzotriazoles). Mean values (n = 3) are shown, standard deviations too small to be shown (below 1%). Solid lines were fitted by a linear function with R^2^ values being >0.99.

**Figure 2:**
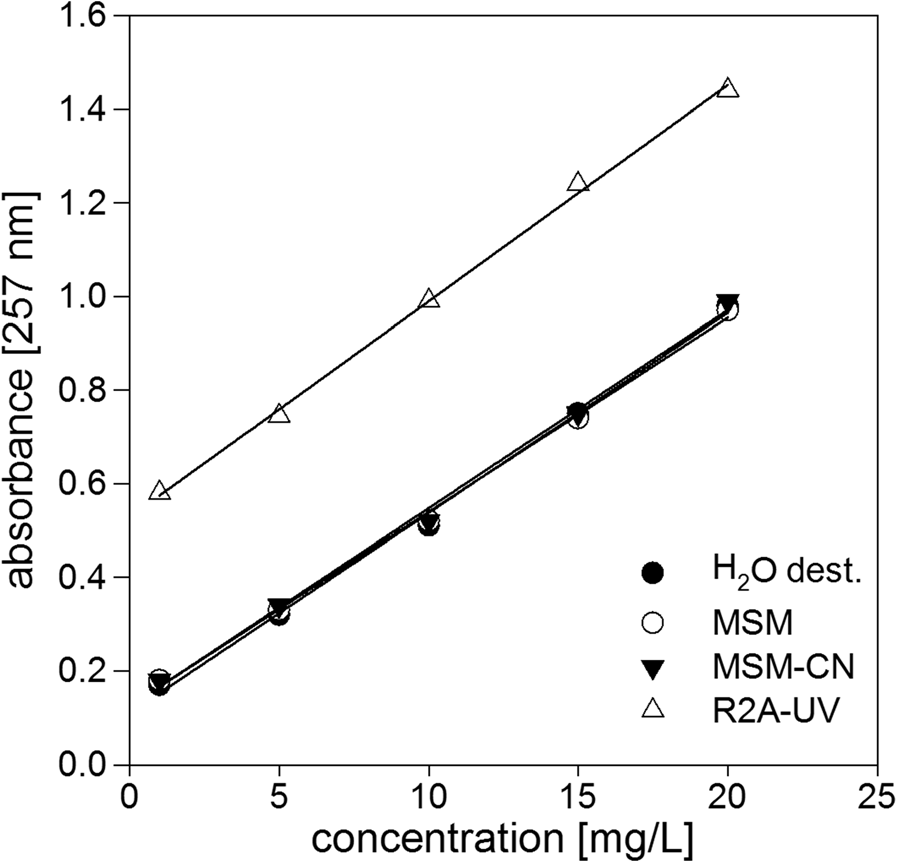
**Effect of used media on UV-absorbance.** Shown are, as all other compounds behaved similarly, calibration curves for SMX in pure water and the three media R2A-UV, MSM-CN and MSM (Table 2). SMX concentrations were 1.0, 5.0, 10.0, 15.0 and 20.0 mg L^-1^. Mean values (n = 3) are shown, standard deviations below 1%. Solid lines were fitted by a linear function with R2 values being >0.99.

The spectra of the selected compounds were taken in high-purity water to find maximum absorbance and to test whether the used concentrations show sufficient absorbance values for reliable measurements (Figure 1A). Calibration followed to evaluate the compounds behavior in plastic microplates and their absorbance values at different concentrations both in high-purity water (Figure 1B) and the used media (Figure 2 for SMX; BTs not shown, as their pattern was the same). Absorbance curves and absorbance maxima were different for the xenobiotic compounds analyzed (Table 1, Figure 1A) and an optimal absorbance range for direct measurements was obtained for concentrations from 2-20 mg L^-1^ for all tested xenobiotics. Maximum absorbance values, correlating with the used concentration of xenobiotic compounds, should lay within 0.2 and 1.0 for optimal measurement according to the plate reader setup. Otherwise dilution or higher sample volumes, to increase thickness, have to be used. Absorbance maxima for the used xenobiotics were evaluated (Table 1) and subsequently used for single wavelength measurements. SMX was measured at 257 nm and benzotriazole, 4- and 5-tolyltriazole at 262 nm (mean value, Figure 1A). It was not possible to screen for biodegradation in setups containing more than one of the tested xenobiotic compounds as their absorbance maxima were almost equal and they thus could not be separately detected in mixed setups (see Figure 1A, BTs). Therefore, only one compound per setup could be monitored. Subsequent calibration was performed using 257 and 262 nm to prove the reliability of UV-AM with different concentrations and different media. SMX and the BTs absorbance values behaved linear related to concentration in pure water (Figure 1B). When tested in different media (Figure 2 for SMX, BTs same behavior, data not shown), the media composition showed no effect on absorbance linearity but increased the background absorbance (Figure 2) in case of R2A-UV as it contains a high amount of complex nutrients. Substrate consumption and growth of biomass influences on UV-AM could be ruled out by relating xenobiotics containing setups with blank setups without xenobiotics. MSM and MSM-CN showed the same low absorbance as high-purity water as they just contained mineral salts. MSM-CN showed a slightly higher background that might be attributed to the higher nutrient concentration. Therefore, detection of all tested xenobiotic compounds was possible in pure water as well as in the used media by UV-AM.

**Table 1:** **Maximum absorbance (abs**_**max**_**) wavelength of tested xenobiotic compounds and corresponding absorbance value for 10 mg L**^**-1**^

**Analyzed compound**	**Absorbance maximum (abs**_**max**_**)[nm]**	**Absorbance value at abs**_**max **_**[10 mg L**^**-1**^**]**
BTri	253/260	0.52
TTri	263	0.49
4-TTri	262	0.48
5-TTri	264	0.49
SMX	257	0.57

### Screening for transformation products

During biodegradation, the molecular structure of the xenobiotic compounds is changed and transformation products might be formed as already shown for SMX [23,26,28]. Thus a change in absorbance could happen that might be observed by spectral scans. Screening for potential transformation products that might exhibit different spectra was performed whenever fast biodegradation was detected, i.e. a significant decrease in absorbance values was observed. For that purpose, unnaturally high concentrations up to 100 mg L^-1^ were tested as transformation products normally appear in very low amounts [29]. Unfortunately, even if it should theoretically be possible to detect transformation products by UV-AM spectral scans, it was, under the given experimental conditions, not possible to screen for transformation products that result from biodegradation of the parent substance. As many transformation products occur in significantly lower concentrations than the parent substances, UV-AM might not be sensitive enough to detect them. A subsequent screening for transformation products with LC/GC methods was also performed and lead to the detection of sulfamethoxazole transformation products (unpublished data). However, a screening for benzotriazole transformation products did not lead to new findings and thus there is urgent need for further research in that area.

### Optimizing media for UV-AM

Measurements at xenobiotic-specific wavelengths and under specific laboratory conditions allowed detection of biodegradation in concentrations above 1.0 mg L^-1^ (Figure 1B) as absorbance values are related to compounds concentration. It was possible to detect relative changes in the compounds concentration but optimizations were necessary as the used media strongly influenced UV-AM in two ways: 1) High background. Especially original R2A media showed high background absorption due to the nutrient composition. 2) Nutrients. Available nutrients led to biomass growth and therefore increased absorbance (Table 2) as at 257 and 262 nm also DNA, cells, and cell-particles show an absorbance [30]. Media containing nutrients like e.g. yeast extract or peptone show high media background absorbance and foster biomass growth but are not well suited for UV-AM as background absorbance can superimpose potential biodegradation. Nevertheless, these media enable growth of a high diversity of organisms and contain complex nutrient sources that may enhance cellular activity and therefore biodegradation. As already shown for SMX, sufficient nutrient availability can foster biodegradation [23].

**Table 2:** Media characterization, xenobiotic application setup and background absorbance at two different wavelengths used for monitoring xenobiotics’ biodegradation

**Media**	**Components [g L**^**-1**^**]**	**pH**	**Xenobiotics’ application setup**	**Absorbance [nm]**	
				**257**	**262**
MSM (minimal salt media)	KH_2_PO_4_ (0.08), K_2_HPO_4_ (0.2), Na_2_HPO_4_ (0.3), MgSO_4_ × 7 H_2_O (0.02), CaCl × 2 H_2_O (0.04), FeCl_3_ × 6 H_2_O (0.003), Hoagland trace elements (0.1 mL L^-1^)	adjusted to 7.4	Xenobiotics as sole C and N source	0.07	0.07
MSM-CN (+ C and N)	As MSM including: sodium acetate (0.5) and NH_4_NO_3_ (0.01)	adjusted to 7.4	Xenobiotics’ co-metabolism	0.08	0.09
R2A-UV (for UV-AM)	Casein peptone (1.0), glucose (0.5), sodium acetate trihydrate (0.5), KH_2_PO_4_ (0.3), soluble starch (0.3), Hoagland trace elements (0.1 mL L^-1^)	adjusted to 7.2	Foster growth of many organisms, xenobiotics’ co-metabolism	0.53	0.56

Therefore original R2A media, being necessarily used for its complex nutrient composition had to be optimized as it showed absorbance values around 3.40 units. The two MSM media already showed very low absorbance values around 0.08 and could be used without changes. The exchange of nutrients in original R2A without changing the total nutrient concentration led to development of R2A-UV media and a decrease in background absorbance from 3.40 to 0.54 units (composition see Table 2, exchanged nutrients in bold). In media or water without biomass, xenobiotics’ concentrations could be measured, but as growth of organisms influences absorbance, exact concentrations’ determination could not be provided with extensive biomass growth in the setup. Therefore, it was necessary to test the influence of biomass on UV-AM. Nevertheless, UV-AM proved applicable for fast and inexpensive detection of relative changes in xenobiotics’ concentrations within the applied media, given the restriction that only one compound can be used per biodegradation setup.

### Screening activated sludge (AS) cultures for xenobiotic biodegradation

#### UV-AM evaluation with biomass

After detection of a decrease in UV-absorbance indicating biodegradation, these setups were analyzed by LC-UV for SMX, and GC-MS/MS for benzotriazoles to validate UV-AM in different matrixes and relate absorbance with exact concentrations. If a screening in biomass-containing setups is possible, UV-AM can significantly increase performance while reducing analytical costs (Table 3).

**Table 3:** Comparison of UV-AM with LC-UV and GC-MS/MS measurements

	**Benzotriazoles**			**Sulfamethoxazole**		
	**UV-AM**	**LC**	**GC**	**UV-AM**	**LC**	**GC**
Time per sample [min]	<1	-	20	<1	5	-
Costs per sample [€]	0.12	-	40–60	0.12	30–40	-
Measurement of biodegradation	Yes	-	Yes	Yes	Yes	-
Measurement of concentrations	Difficult	-	Yes	Difficult	Yes	-
Labor intensive	No	-	Yes	No	Yes	-
Time intensive	No	-	Yes	No	Yes	-
Monitoring biodegradation	Yes	-	Yes	Yes	Yes	-

Compared are time needed for analyzes, costs per sample and positive and negative aspects of each analysis technique.

**Figure 3:**
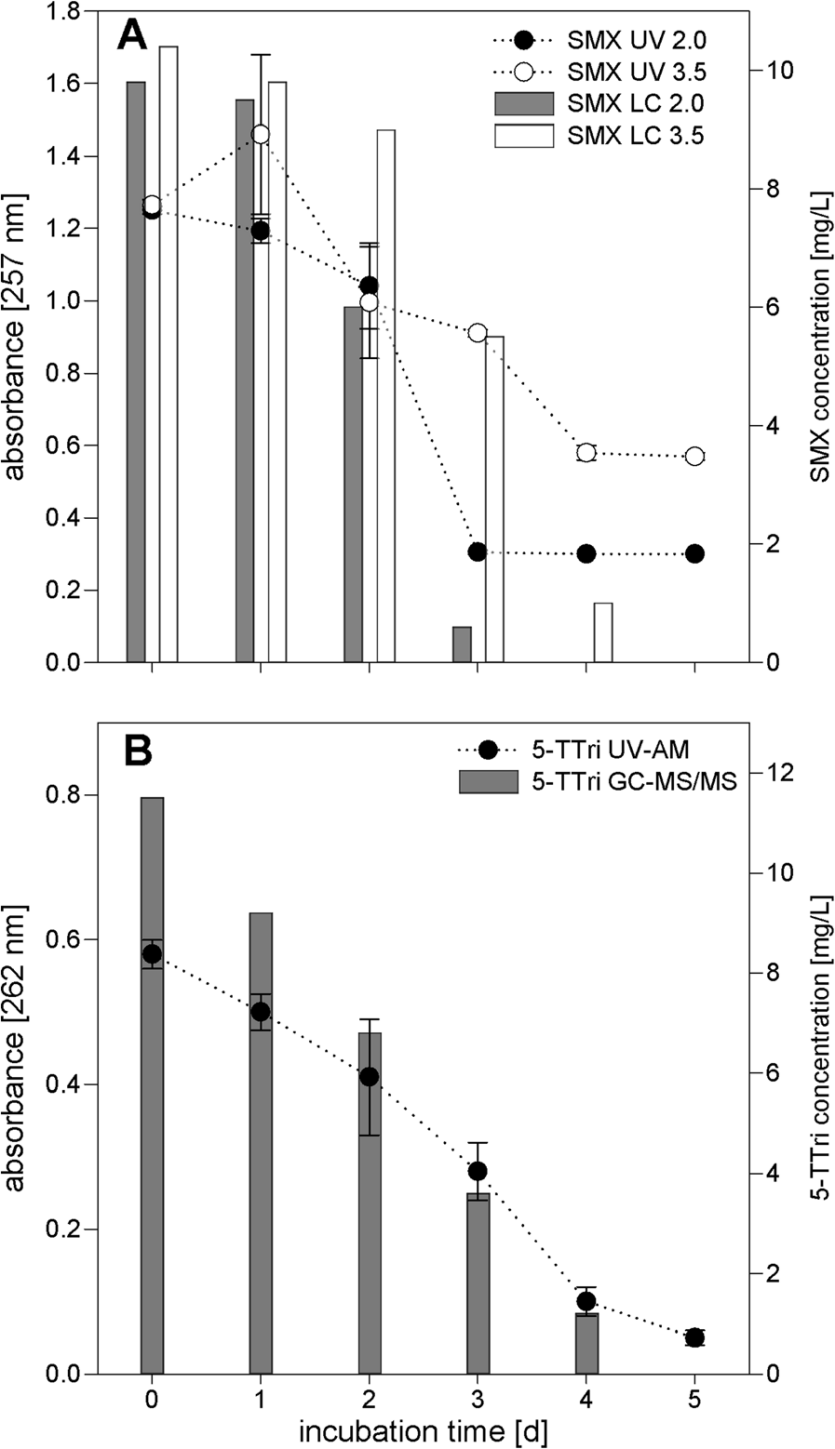
**A and B Correlation of UV-AM results with LC-UV and GC-MS/MS analyses. A)** Shown is SMX biodegradation in R2A-UV media with two different MLSS concentrations (2.0 and 3.5 g L^-1^ MLSS). Initial SMX concentration 10 mg L^-1^. Dotted lines show the measured SMX biodegradation detected with UV-AM, columns represent SMX concentration measured with LC-UV. **B)** The same is shown for 5-TTri (initial concentration 10 mg L^-1^, measured with GC-MS/MS) biodegradation with initial MLSS concentration of 0.5 g L^-1^. Shown are mean values of duplicate experiments with error bars indicating standard deviations (n = 2) (only for UV-AM).

All AS communities were able to biodegrade most of the tested compounds under aerobic conditions in R2A-UV media, regardless the initial MLSS concentration. Degradation was observed with xenobiotic concentrations of 10 mg L^-1^ and it was possible to compare different initial biomass concentrations. Incubation times until degradation was detectable with UV-AM varied with the tested compound and initial biomass concentration. Chosen setups were analyzed in a biodegradation time series by both UV-AM and LC-UV or GC-MS/MS to test the reliability of UV-AM results (Figure 3). Double measurements using UV-AM and LC/GC methods proved that SMX as well as benzotriazole biodegradation could be detected by UV-AM as the decrease in absorbance values could be correlated to the declined SMX and benzotriazoles’ concentrations determined by LC and GC measurements. Even in experiments with a high biomass concentration of 3.5 g L^-1^ MLSS (Figure 3A), the decrease in UV-AM fitted the measured values in LC-UV, but the background was increased significantly probably due to solved compounds originating from the AS inoculum (see Figure 3A, at day five background absorbance of 0.56 units with all SMX being biodegraded). In contrast, the 5-TTri setup carried out with only 0.5 g L^-1^ MLSS (Figure 3B) after 5 days of incubation showed hardly any background absorption. Therefore, this lower MLSS concentration seems to be more useful for UV-AM. Nevertheless, high MLSS concentrations, as they are used in WWTPs, had to be tested to evaluate if UV-AM can also be used directly for screening activated sludge communities for their biodegradation potential. Even with high biomass density, a proper sedimentation provided and sorption of the target compounds onto biomass being neglected, UV-AM represented a fast and cost-effective way to screen for biodegradation (Table 3). Screening 96 samples with UV-AM, including sample preparation, took around 30-40 min and required costs of around 12 €. Compared to LC-UV or GC-MS/MS this is a significant saving in time and money when concentrations are high enough in laboratory expermiments.

### UV-AM for detection of xenobiotics’ biodegradation under different nutrient conditions

**Figure 4:**
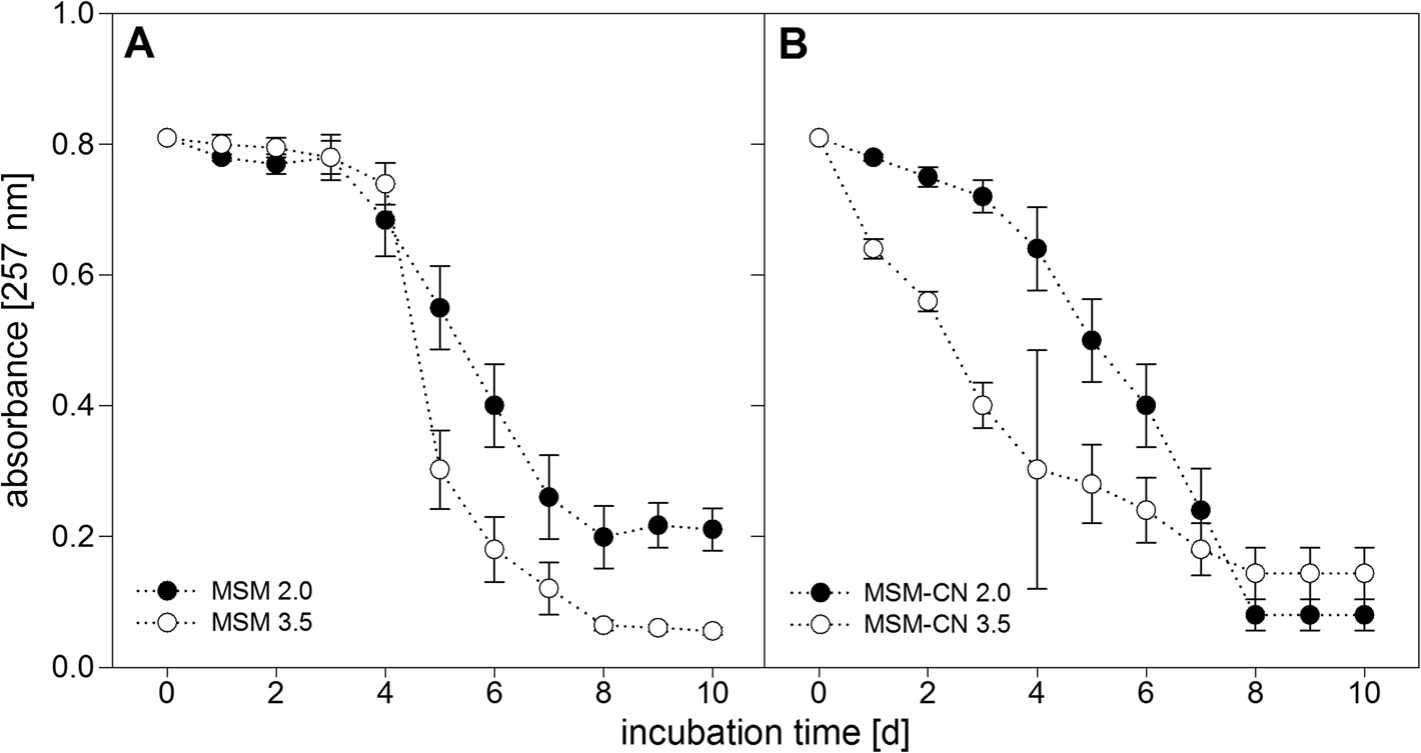
**A and B Time series of SMX biodegradation with activated sludge-communities as detected by UV-AM. A)** in MSM-CN and **B)** in MSM media**.** Initial SMX concentration 20 mg L^-1^. Shown are mean values of SMX absorbance in duplicate experiments with error bars indicating standard deviations (n = 2).

Another advantage of UV-AM is the possibility to screen for differences regarding nutrient concentration and biomass concentration. Figure 4A and B show for SMX (benzotriazole not shown as it behaved similarly) two setups differing in nutrient concentration (MSM or MSM-CN media) and biomass amount (2.0 or 3.5 g L^-1^ MLSS). It became clear that in MSM-CN biodegradation did not require any acclimatization, it started right after inoculation, presumably due to the microorganisms’ higher activity and showed a more constant and linear removal. In MSM an acclimatization period of almost four days was observed followed by a very rapid SMX removal within four days. Another finding was that a higher biomass concentration positively affected biodegradation as setups with 3.5 g L^-1^ MLSS worked slightly better in both duplicates. This effect was rather clear for MSM-CN but with nutrients shortage in MSM, biomass concentration did not have that effect on biodegradation as the general activity was lower and thus more time for acclimatization required. In both setups SMX was biodegraded in around 8 days. Additional experiments were performed regarding nutrient composition and initial biomass concentration (Table 4). Only initial xenobiotics’ concentrations were measured by LC/GC and a large number of setups were screened for their biodegradation potential. As Table 3 also shows, a lot of setups could not be measured as background absorption was too high for detection of changes in absorbance values. Especially setups with 1 mg L^-1^ initial xenobiotics’ concentration were superimposed by higher background absorbance due to biomass concentration.

**Table 4:** Biodegradation and time needed for absorbance decrease in the different activated sludge community setups using different media, biomass concentration and initial xenobiotics’ concentrations

**Xenobiotic**	**Concentration[mg L**^**-1**^**]**	**Initial MLSS [g L**^**-1**^**]**	**Decrease of absorbance values**^**1**^			**Lowest absorbance value achieved after [d]**^**2**^			**Biodegradation confirmed by LC /GC**
			**R2A-UV**	**MSM-CN**	**MSM**	**R2A-UV**	**MSM-CN**	**MSM**	
BTri	1	0.5	✓	✓	✓	10	12	13	Yes
		2.0	n.d.	n.d.	n.d.	n.a.	n.a.	n.a.	No
		3.5	n.d.	n.d.	n.d.	n.a.	n.a.	n.a.	Yes
	10	0.5	✓	✓	✓	15	16	18	Yes
		2.0	✓	✓	✓	14	12	13	Yes
		3.5	n.d.	n.d.	n.d.	n.a.	n.a.	n.a.	Yes
5-TTri	1	0.5	✓	✓	✓	10	8	7	Yes
		2.0	n.d.	n.d.	n.d.	n.a.	n.a.	n.a.	No
		3.5	n.d.	n.d.	n.d.	n.a.	n.a.	n.a.	Yes
	10	0.5	✓	✓	✓	10	9	9	Yes
		2.0	✓	✓	✓	8	8	8	Yes
		3.5	n.d.	n.d.	n.d.	n.a.	n.a.	n.a.	Yes
SMX	1	0.5	✓	✓	✓	4	3	4	Yes
		2.0	n.d.	n.d.	n.d.	n.a.	n.a.	n.a.	No
		3.5	n.d.	n.d.	n.d.	n.a.	n.a.	n.a.	No
	10	0.5	✓	✓	✓	6	5	8	Yes
		2.0	✓	✓	✓	3	8	8	Yes
		3.5	✓	✓	✓	4	8	8	Yes

Shown are BTri, 5-TTri and SMX. 4-TTri was not biodegraded and is therefore not shown.

n.d. – biodegradation determination not possible, background due to biomass concentration too high.

n.a. – not applicable as no biodegradation could be detected by UV-AM.

^1^Change in UV-AM indicates biodegradation.

^2^Time needed to achieve a stable value in UV-AM after a change in absorbance.

## Conclusions

A UV absorbance measurement technique was developed as a pre-test for the evaluation of xenobiotics’ biodegradation potential. UV-AM proved time- and cost-saving, reproducible and reliable to screen a large number of different laboratory setups within short time, given the premise that the concentrations of the tested compounds are within the range of 1.0 to 20.0 mg L^-1^. Additionally, specific media were used to reduce both, background absorption and biomass growth. As a limitation only one compound could be used per biodegradation setup, e.g. benzotriazole, 4- and 5-tolyltriazole or sulfamethoxazole could be analyzed by UV-AM to identify biodegradation potential. It was possible to detect xenobiotic biodegradation in reactors inoculated with activated sludge. Validation of UV-AM was performed by comparing the values with either LC-UV for SMX or GC-MS/MS for benzotriazoles. Due to the use of microplates an “easy to handle” system allowing high throughput screening was established. 96 well or even 384 well plate formats can be used, requiring less time and saving laboratory costs. Most important, it is possible to pre-select biodegrading communities, optimize the conditions for biodegradation with respect to nutrient concentration and activated sludge inocula and monitor biodegradation over time and to screen pure culture setups for their biodegradation potential. This minimizes required time and costs for LC or GC measurements as only setups showing a decrease in absorbance need to be further analyzed and can be used in subsequent biodegradation experiments. However, high biomass concentrations turned out disadvantageous as they created a high background absorbance and thus superimposed UV-AM. Furthermore, only relative changes in xenobiotics’ concentrations could be detected which is no big problem as for pre-selections or monitoring approaches exact concentrations are not needed.

## Methods

### Chemicals and glassware

1-H-benzotriazole (BTri) and tolyltriazole (TTri), consisting of 4-tolyltriazole (4-TTri) and 5-tolyltriazole (5-TTri) (40%/60%, w/w), were provided by Cimachem GmbH (Kirchheimbolanden, Germany). 5-TTri, 4-TTri (as separate compounds) and sulfamethoxazole (SMX) were obtained from Sigma Aldrich (Steinheim, Germany) as well as sodium carbonate solution and toluene. All other chemicals were purchased from Merck KGaA (Darmstadt, Germany). High-purity water used for media, solutions, buffers, and analyses was prepared by a Milli-Q system (Millipore, Billerica, MA, USA). All used glassware was from Schott AG (Mainz, Germany) and pre-cleaned by an alkaline detergent (neodisher®, VWR Darmstadt, Germany) followed by autoclaving for 20 min at 121°C.

### Activated sludge (AS)

AS was taken from biological stage 1 of a 2-stage municipal conventional activated sludge-plant (CAS-M) treating 1 million populations equivalents. The influent consists of municipal as well as up to 50% industrial wastewater. 500 mL AS were collected for reactor inoculation in pre-cleaned 1L glass bottles, stored at 4°C and used within 24h.

### Experimental setup

#### Inocula and media

Biodegradation capabilities of AS towards BTs and SMX were tested in separate setups. Fresh AS was centrifuged (10 min, 4000 g), the supernatant discarded and the remaining biomass washed with 1xPBS-Buffer (NaCl (8.0 g L^-1^), KCl (0.2 g L^-1^), Na_2_HPO_4_ (2.7 g L^-1^), KH_2_PO_4_ (0.2 g L^-1^)). The procedure was repeated twice to remove wastewater residues. 20 mL of one of the three liquid media (Table 2) were inoculated to an initial concentration of 0.5 g L^-1^ MLSS (mixed liquor suspended solids), 2.0 g L^-1^ MLSS and 3.5 g L^-1^ MLSS, respectively. These media, different in nutrient composition, were used for detection of BTs and SMX biodegradation by UV-absorbance measurements (Table 2). Media were adapted to fit the needs of UV-AM and determine variations in BTs and SMX biodegradation concerning availability of nutrients, degradation rate and test reliability of UV-AM.

### Batch tests

20 mL reactors, inoculated to one of the mentioned MLSS concentrations, were set up in parallel. Each media was spiked with BTri, 4-TTri, 5-TTri and SMX at concentrations of 1, 10, 25, 50 and 100 mg L^-1^. Experiments were performed with R2A-UV media for optimal growth conditions, MSM-CN for testing the need of carbon/nitrogen and MSM for highly selective growth conditions due to nutrient depletion. Concentrations of BTs/SMX were varied to ensure acclimatization and increased selectivity. In total 630 experiments were set up (7 compounds, 5 concentrations, 3 media, 3 different MLSS concentrations, each in 2 parallels).

All experiments were carried out in the dark to avoid photolysis. Aerobic conditions were ensured by shaking the reactors at 150 rpm on an orbital shaker. Temperature was kept constant at 20°C (± 2°C), pH controlled to be in the range of 7 to 8.

All experiments were performed until absorbance reached a stable and distinctly lower value compared to the initial one. In case no change in absorbance was detected, the experiment was stopped after a maximum of 56 days.

### Sampling and sample pre-treatment

Sampling of the reactors and detection of biodegradation by UV-AM was carried out once a day by withdrawing 1 mL supernatant from each reactor after 30 min sedimentation to reduce biomass withdrawal and media turbidity. 200 μL for UV-AM and 800 μL for both duplicate measurements and LC-UV (SMX) or GC-MS/MS (BTs) analyses were needed. Samples for UV-AM were analyzed the same day or could be stored frozen at -20°C until analysis.

### Analyses of SMX and BTs

#### UV-AM microplate test system

200 μL were taken from the reactors and directly used for measurement. Centrifugation (10 min, 8,000 g, 20°C) and/or filtration (0.25 μm, PTFA syringe filter) were applied in case of high cell density (high turbidity) to remove cells and/or cellular debris. Analyses were performed in 96 well plates with a UV-permeable bottom foil (lumox® multiwell, Sarstedt AG, Nürnbrecht, Germany) using an automated plate reader (EnSpire® Multimode Plate Reader, Perkin Elmer, Rodgau, Germany). UV spectra of all used compounds from 230 to 330 nm at 10 mg L^-1^ in pure water and the three used media were recorded and absorbance maxima of the substances determined. Calibration was carried out for all tested compounds with 0.1, 1.0, 5.0, 10.0 and 20.0 mg L^-1^ in pure water and used media. Background absorbance of used 96 well UV-plates filled with 200 μL high-purity water (=working volume) was 0.07– 0.09 units. Linear absorbance, due to the internal calibration of the reader itself, was ensured when the absorbance lay within a range of 0.2 to 1.0 absorbance units. For each measurement a blank (media and xenobiotic, without biomass) was measured to detect changes over time as well as a zero blank (only media) to detect background absorbance.

### GC-MS/MS and LC-UV analyses

Samples from the reactors were centrifuged (10 min, 8000 g, 20°C), filtered through an 0.45 μm PTFE filter, filled into sterile glass flasks for LC/GC, and stored in the dark at -20°C upon analysis.

LC-UV measurements were done with a Dionex 3000 series HPLC system (Dionex, Idstein, Germany), equipped with an autosampler. DAD scanning from 200 to 600 nm was done to detect and quantify SMX. Limit of quantification and limit of detection were 0.1 mg L^-1^ and 0.03 mg L^-1^, respectively.

Chromatographic separation was performed on a Nucleosil 120 - 3 C18 column (250 mm × 3.0 mm i.d., 3 μm particle size) from Macherey Nagel (Düren, Germany). Column temperature was 25°C. The mobile phases were acetonitrile (AN) and water (pH 2.5 using phosphoric acid). The gradient used was 0-5 min, 7% AN; 5-18 min, 7-30% AN; 18-30 min, 30% AN; 30-35 min, 7% AN. The solvent flow rate was 0.6 mL/min. The column was allowed to equilibrate for 5 min between injections.

For GC-MS/MS measurements, chemical preparation and determination of the BTs’ concentration in the liquid samples were performed according to Liu et al. (2011), with the following steps: all samples were derivatised with acetic anhydride and extracted with toluene. The calibration curve was determined with aqueous solutions containing 0.1, 1.0, 6.25, and 25 mg L^-1^ of the three analytes BTri, 4-TTri, and 5-TTri. These three solutions, high-purity water serving as a blank, and the prepared samples were spiked with an internal standard solution (5,6-dimethyl-benzotriazole in sodium carbonate solution) containing the same concentration as used for the calibration curve. The extracts were analyzed by GC tandem mass spectrometry (GC-MS/MS) on a Saturn 2200, Varian (Agilent Technologies Deutschland GmbH, Böblingen, Germany) equipped with an ion trap. Limit of quantification and limit of detection for this setup were 0.1 μg L^-1^ and 0.01 μg L^-1^, respectively. Compound separation was accomplished on a VF-5ms column from Varian (30 m × 0.25 mm, film thickness 0.25 μm) perfused by helium as the carrier gas at a constant flow rate of 1.5 mL/min. The temperature profile started at 65°C (held for 4 min), was increased by 12°C/min to 200°C, and was finally set to 300°C at a rate of 40°C/min (held for 6 min). Operation mode of the MS/MS was resonance excitation of the characteristic precursor ions of the analytes and the internal standard. Injection was performed splitless ranging from 1.0 to 9.0 μL sample volume (large volume liner, Varian 1079 programmable injector). Blanks were analyzed to check for possible contaminations of the experimental samples and to verify the accuracy of the method itself.

## Competing interests

The authors declare that there are no competing interests concerning this manuscript.

## Authors’ contributions

BH drafted the manuscript, designed and carried out the biodegradation experiments. HL reviewed the manuscript. HH and EM conceived of the study, participated in its coordination and helped to review the manuscript. All authors read and approved the final manuscript.
